# Validation of the Child-Oral-Health-Impact-Profile among adolescents in Johannesburg: A cross-sectional study

**DOI:** 10.4102/phcfm.v15i1.3993

**Published:** 2023-10-25

**Authors:** Yolanda Malele-Kolisa, Innocent Maposa, Veerasamy Yengopal, Jude Igumbor

**Affiliations:** 1 Department of Community Dentistry, Faculty of Health Sciences, University of the Witwatersrand, Johannesburg, South Africa; 2 School of Public Health, Faculty of Health Sciences, University of the Witwatersrand, Johannesburg, South Africa

**Keywords:** oral health-related quality of life, adolescents, HIV, self-rated-oral-health, untreated caries, patient-reported-outcomes

## Abstract

**Background:**

Oral health-related quality of life (OHRQol) is described as the effect of oral conditions on the overall functioning and well-being of individuals.

**Aim:**

This study sought to determine the validity of a modified-child oral health impact profile (M-COHIP) among adolescents living with the human immunodeficiency virus (HIV) infection (ALHIV) and HIV-undiagnosed adolescents and establish the factors influencing OHRQoL among adolescents in central Johannesburg.

**Setting:**

Schools and HIV wellness centre in central Johannesburg.

**Methods:**

An interviewer-administered questionnaire was applied, followed by an oral examination.

**Results:**

A total of 504 adolescents were included in the study. The overall mean decayed teeth for permanent dentition was 1.6 (standard deviation [s.d.]: 1.99) and caries prevalence was 62.2% (*n* = 309). The tool’s Cronbach’s alpha was 0.88. The item-rest correlations were from 0.6 to 0.85 for all items. The initial exploratory factor analysis explained 76% of the total variance. The overall M-COHIP score was 59.6 (18.2). The overall modified-COHIP scores for those not in care (schools) were higher [62.88] than those of ALHIV. The poor M-COHIP scores were associated with reporting toothache, having active decay, poor oral health-self-rating, and being selected from the school site (*p* < 0.005).

**Conclusion:**

The validation study supports the use of the tool as a reliable and valid measure of OHRQoL. Future research can investigate the extent to which the tool is effective in measuring treatment outcomes and patient satisfaction.

**Contribution:**

The validated tool will be beneficial in the African context for programme assessments and overall measure of quality-of-life impacts from oral conditions.

## Introduction

The adolescent phase of life involves a transition from childhood to adulthood. This transition is characterised by a multitude of physical, emotional, psychological and developmental changes. Health changes and health concerns may also be unique to this phase in life – specifically, oral health which is an integral part of overall health and well-being.^1^ The literature on adolescent oral health underscores the increasing dental caries burden and higher oral health needs.^2^ The oral disease burden does affect the oral health related quality of life (OHRQoL). In African settings, adolescents and adolescents living with the human immunodeficiency virus (HIV)-infection (ALHIV) have been marginally investigated in the broad field of OHRQoL.^3^ The ALHIV constituted about 1.6 million of all people living with HIV in 2012. South Africa carries the highest burden of ALHIV and accounts for about 310 000 of the global burden.^4^ Adolescents living with HIV on antiretroviral therapy face unique challenges in maintaining oral health. Environmental factors such as diet, self-image disturbance, and social acceptance can further exacerbate oral lesions. It is important that considerations for managing oral health among this patient population are studied.^5^

Oral health-related quality of life is the effect of oral conditions on the overall functioning and well-being of individuals.^6^ The OHRQoL is related to how people grow, look, speak, taste, socialise, and their perceptions of social well-being^7^ and can impact the daily activities of adolescents.^6^ These daily activities are essential elements of the formative years of adolescents. According to Barbosa and Gaviao, socially and contextually responsive tools are required to assess OHRQoL.^8^ The children in different geographical regions have responded differently to the social and emotional well-being constructs of OHRQoL tools.^9^ The difference can be explained by the sociocultural way of life in the current context.^8^

The OHRQoL measures were developed in various settings other than Africa even though some have been translated and validated in KiSwahili-Tanzania^10^ and Afrikaans^11^ in Africa. For these tools to be applied in the South African setting they may have language and contextual limitations leading to bias.^12^ Adaptation and concomitant validation are thus preferred for reliable assessment of OHRQoL in a local setting.

There are various tools used to measure the OHRQoL and the Child Oral Health Impact Profile (COHIP) instrument, developed by Broder et al., is an OHRQoL measure incorporating both negative and positive health impacts.^13^ It has 19 items with five dimensions: *oral health, functional well-being, socio-emotional well-being, school performance and self-image.* This study added three dimensions: *dental care access, dental care experiences and coping theme*, (with 10 items) based on the initial published qualitative inquiry,^14^ which was a part of a larger study. This study, therefore, is part of a larger study and seeks to establish the validity of this modified measure of the OHRQoL of HIV-infected and HIV-undiagnosed adolescents in the South African setting.

## Research methods and design

### Study design

A cross-sectional study design was used for the study.

### Study setting

The study was conducted at the HIV Wellness Unit of the Paediatric Virology Unit at the Charlotte Maxeke Johannesburg Academic Hospital. The hospital is a tertiary referral centre for the catchment population of the Greater Johannesburg metropolitan area and the periphery. The Department of Community Dentistry, sees weekly, children and adolescents and provides dental screening, preventive and relief of pain and sepsis services.

### Study participants

The participants were the ALHIV attending the wellness site, aged 10–19 years. They were recruited and enrolled as they came for their daily wellness visits in February–June 2018. The conditions for entry into the study were the adolescents’ parental consent and their own assent following informed consent. A comparator group was the adolescents attending public schools in the inner city of Johannesburg.

The school participants were recruited from the two public schools within the department’s community outreach projects. They were randomly sampled from a sampling frame of 400 learners in Grades 7–12, aged 11–19 years. The exclusion criteria for the school participants were those who reported medical history, those on chronic medication, and those without parental consent forms and learners’ assent forms. These participants were not tested for HIV and thus were considered HIV-undiagnosed.

Similarly, the sample of 226 ALHIV were conveniently recruited from the HIV-Wellness Centre. No matching was intended. Challenges mainly logistically related to seeking care and recruitment at the wellness centre impeded getting the expected sample size of 266. The participants were patients who came in for their routine wellness services at the centre. All adolescents, with written informed assent and parental consent, were eligible to be participants. The study received ethical approval from the Human Research Ethics Committee of the University of Witwatersrand, no. M161142.

### Sample size estimation and sampling procedure

The sample size was calculated based on the 400 population in school, by assuming the error margin of 5%, with a 95% confidence interval. Study sample size calculation suggested 280 randomly selected school learners for the comparator group and 266 for the ALHIV group. A convenience sampling was used where the participants formed at the sample following recruitment.

### Data collection

Data were collected during healthcare routine consultations. Dental examinations were performed by two calibrated dental practitioners (Y.M.-K., Pumla S.). A dental clinical examination was based on the Decayed Missing and Filled Teeth (DMFT) index as outlined in the World Health Organization survey methods. The Oral HIV/AIDS Research Alliance (OHARA) case definitions were used to record the oral mucosal conditions for the participants at the wellness centre.^15^ The inter-examiner reliability was performed by re-examining one-tenth of the sample by each examiner, and the final kappa statistics was 0.81 for DMFT, and 0.87 for OHARA case definitions.

### Modified-child oral health impact profile administration

After the clinical examination, the 29-item modified-child oral health impact profile (M-COHIP) was interviewer-administered in English to all participants and explanations were carried out in the language of preference. The following were the responses required from the participants: *never, almost never, sometimes, fairly often, all the time* had the event occurred in the past 3 months, attributed to one of the 29 items. The self-rated oral health responses expected from the question ‘how would you rate the health of your teeth and mouth in the past 3 months’ ranged from *poor, fair, average, good and excellent*.

### Data analysis

Descriptive statistics for socio-demographic, decayed scores, DMFT and M-COHIP scores were calculated. Bivariate analysis was performed through the comparison of all scores (dichotomized as D = 0 and D > 0 and DMFT = 0 and DMFT > 0).

The M-COHIP score was calculated by adding the answer responses, which ranged from 1 to 145, with higher scores implying a poorer OHRQoL. Cronbach’s alpha was used to determine the internal consistency of the scale. Items were added, removed and modified, according to whether the indices of reliability improved.^16^

The construct validity was assessed by the measures of discriminant validity using Bonferroni post hoc test and *t*-tests. The following hypothesis was assumed: participants with a higher M-COHIP score will have poorer self-rated oral health. Convergent and discriminant validity assessments were also performed based on M-COHIP scores and overall oral health self-rating, toothache and active dental caries (D > 0). Multiple logistic regression was performed to calculate the predictors of M-COHIP adjusting for sex, age and research site.

The orthogonal rotated pattern matrix using factor loadings of 0.4 and above was conducted and resulted in five factors with M-COHIP 29 items and 504 participants. The Kaiser-Meyer-Olkin Measure showed sampling adequacy of KMO = 0.870 while Bartlett’s tests of sphericity was χ^2^ (406) = 2728.63, *p* < 0.001 and revealed correlations between items were large enough for exploratory factor analysis (EFA). The initial EFA performed on all items explained 76% of the total variance and showed two factors with eigenvalues over one. The plot from the parallel analysis revealed six factors. However, this study adopted a five-factor loading based on similar studies^17^ to determine latent factors to be retained and consistent with the original Broder tool.

Based on the theoretical model of OHRQoL, the relationships between the three latent factors, viz. individual, external level factors and social impact level factors were explored using the confirmatory factor analysis (CFA). The multiple fit indices were used to determine the CFA model fit. The root mean square error of approximation (RMSEA), Akaieke’s information criterion (AIC), Bayesian information criterion (BIC), Tucker–Lewis Index (TLI), comparative fit index (CFI) and finally the standard root mean square residual (SRMR) were used. The values greater than 0.95 imply an excellent fit while 0.90 an acceptable fit. The SRMR acceptable fit should be less than 0.10 while the RMSEA value indicative of close fit is less than 0.05.

### Ethical considerations

Ethical clearance to conduct this study was obtained from the University of the Witwatersrand Human Research Ethics Committee (Medical) (No. M161142).

## Results

### Dental caries prevalence and HIV-oral lesions

The response rate was good at 99%; all the recruited participants at wellness centre and 278/280 in schools answered the questionnaire interviews. The overall mean decayed teeth for permanent dentition was 1.6 (standard deviation [s.d.]: 1.2), and the overall caries prevalence was a high 62.2 % among all adolescents. When categorised by group, the adolescents in the HIV clinic had lower caries prevalence of 53.7% than those in school at 66.2%, but higher mean decayed teeth 2.0 (s.d.: 2.56) versus 1.2 (s.d.: 1.26), (*p* ≤ 0.05). The ALHIV selected from the wellness site displayed higher treatment component through the filled (F), and the extracted (M) teeth mean scores. In the wellness group, oral mucosal lesions prevalence – denoted by OHARA > 0 (at least one soft tissue lesion), was 21.7% (Table 1).

**TABLE 1:** Socio-demographics characteristics and caries occurrence, modified-child oral health impact profile scores of the participants by the research group.

Trait	HIV wellness site; *n* = 226				School site; *n* = 278			
	*n*	%	Mean	s.d.	*n*	%	Mean	s.d.
**Age (Min to Max 11–20 years)**	-	-	15.14	2.10	-	-	15.10	1.94
Age 11–15 years old	132	58.4	-	-	125	45.0	-	-
Age 16–20 years old	94	41.6	-	-	153	55.0	-	-
Total	226	-	-	-	278	-	-	-
**Gender**								
Male	103	47.0	-	-	195	70.7	-	-
Female	116	53.0	-	-	81	29.3	-	-
Total	219	†	-	-	276	†	-	-
**Employment**								
Self-employed†	34	19.3	-	-	105	40.4	-	-
Employed parent†	77	43.8	-	-	116	44.6	-	-
Unemployed parent†	65	36.9	-	-	39	15.0	-	-
Total	176	†	-	-	260	†	-	-
**Schooling**								
Primary school†	43	18.7	-	-	103	37.3	-	-
High school†	175	80.3	-	-	173	62.7	-	-
Total	218	†	-	-	276	†	-	-
Decayed = 0†	96	42.7	-	-	92*	33.8	-	-
Decayed > 0†	129	57.3	-	-	180*	66.2	-	-
Total	225	†	-	-	272	†	-	-
OHARA = 0	177	78.3	-	-	-	-	-	-
OHARA > 0	49	21.7	-	-	-	-	-	-
Total	226	-	-	-	-	-	-	-
D	-	2.56	2.0	2.56	-	-	1.2***	1.26
M	-	-	0.6	1.09	-	-	0.2***	0.44
F	-	-	0.7	0.85	-	-	0.3***	0.89
DMFT	-	-	3.4	2.07	-	-	1.6***	1.64
**Sub-scales and items**								
Confidence interval								
Total M-COHIP score	-	-	55.5	18.05	-	-	63.6**	17.42
Min–max 14–115	-	-	53.1–57.9	-	-	-	61.1–66.1	-

M-COHIP, modified-child oral health impact profile.

s.d., standard deviation.

* , *p* ≤ 0.05;

** , *p* < 0.01;

*** , *p* ≤ 0.001.

† , missing values.

### Modified-child oral impact score

The overall M-COHIP score was 59.6 (18.42), median 58 (interquartile range [IQR]: 46 to 72). The lowest score was 14 and highest was 115. When reported by site, the HIV-wellness group score was 55.5 (18.05), while that of the school was 63.6 (17.42) (Table 1). The overall Cronbach’s alpha was 0.88 for the 29 items. The five sub-scales in the Broder COHIP-SF tool were retained, and three new sub-scales were added to calculate the reliability of the scale. All eight sub-scales had reliability ranging from 0.5 to 0.8 (Table 2). ‘School Performance’ had the lowest scale reliability coefficient of 0.50.

**TABLE 2:** Mean M-COHIP scores and sub-scales score by research site.

Sub-scales and Items	HIV wellness site (*N* = 226)			School site (*N* = 278)		
	Mean	s.d.	Confidence Interval	Mean	s.d.	Confidence Interval
Total M-COHIP score	55.5	18.05	53.1–57.9	63.6	17.42**	61.1–66.1
Oral health score	9.9	4.09	9.3–10.5	11.3	3.91**	10.7–11.8
Self-image score	5.9	2.72	5.6–6.3	6.5	2.44*	6.2–6.9
Social emotional wellbeing score	11.7	5.09	10.9–12.4	13.8	5.39**	12.9–14.5
School performance score	3.2	1.7	2.9–3.4	3.6	1.9*	3.3–3.9
Functional wellbeing score	7.4	3.19	6.9–7.8	8.8	3.34**	8.2–9.2
Dental access score	7.7	4.26	7.2–8.3	7.6	4.26	6.9–8.2
Dental care experience score	5.2	2.64	4.9–5.6	5.6	2.72	5.2–5.95
Coping score	6.5	2.91	6.1–6.9	7.2	3.26*	6.7–7.7

** , *p* ≤ 0.001;

* , *p* ≤ 0.05.

s.d., standard deviation.

Most items^10^ loaded on the first component named ‘Social-Emotional Well-being’. The second component had five items and constituted the ‘Functional well-being’ sub-scale. Four items did not load anywhere with the said cut-off points of < 0.4 (Table 3). The item-rest correlations were 0.79 to 0.85 for all items. The item-rest correlation indicated the correlation between an item and the whole scale as formed by all items. The Alpha, if the item is deleted, showed that sub-scale reliability becomes poor when the item is deleted except the item in question (Table 4).

**TABLE 3:** Rotated Pattern Matrix and unique variances of the five-factor component for the modified-child oral health impact profile-29 in South Africa.

Items in the modified COHIP tool	Components					Uniqueness
	Social-emotional well-being	Functional well-being	Dental access	Coping	Self-image	
1. Had pain in your teeth or toothache	0.232	0.413	0.073	0.312	−0.056	0.670
2. Had crooked teeth or spaces between your teeth	0.226	0.308	0.080	0.208	0.127	0.788
3. Had discoloured teeth or spots on your teeth	0.414	0.100	0.234	0.053	0.108	0.749
4. Had bad breath	0.415	0.152	0.094	0.277	−0.034	0.718
5. Had bleeding gums	0.169	0.356	0.010	0.354	−0.002	0.719
6. Been unhappy or sad	0.424	0.251	−0.020	0.297	0.184	0.635
7. Missed school for any reason because of your teeth	0.241	0.619	0.129	−0.042	−0.057	0.538
8. Been confident because of your teeth and mouth	−0.064	−0.001	−0.066	0.010	−0.561	0.676
9. Had difficulty eating foods you would like to eat	0.149	0.493	0.017	0.374	0.019	0.595
10. Felt worried or anxious	0.552	0.320	0.026	0.219	0.063	0.540
11. Not wanted to speak or read out loud in class	0.469	0.214	0.170	0.087	0.175	0.667
12. Avoided smiling or laughing	0.560	0.180	0.162	0.232	0.114	0.561
13. Had trouble sleeping	0.2584	0.636	0.071	0.121	−0.032	0.508
14. Been teased, bullied	0.474	0.141	0.089	0.056	0.040	0.743
15. Felt that you were attractive	−0.008	0.029	−0.066	−0.050	−0.583	0.652
16. Felt that you look different	0.512	0.054	0.158	0.048	−0.132	0.690
17. Had difficulty saying certain words	0.401	0.231	0.117	0.132	−0.165	0.728
18. Had difficulty keeping your teeth clean	0.223	0.141	0.184	0.234	0.232	0.788
19. Been worried about what other people think	0.567	0.028	0.225	0.142	0.103	0.596
20. Had a problem with getting dental care because the clinic or hospital is far	0.180	0.044	0.690	0.247	0.058	0.425
21. Had a problem with getting dental care because my parents or caregivers are usually at work	0.232	0.090	0.608	0.307	0.015	0.474
22. Had a problem with getting dental care because my family cannot afford	0.344	0.065	0.558	0.115	−0.024	0.552
23. Had a problem with getting dental care because the clinics are closed	0.182	0.149	0.563	0.090	0.126	0.604
24. At the dental clinic, they do not take my teeth and mouth complaints seriously	0.038	0.386	0.429	0.006	0.116	0.652
25. At the dental clinic, they do not address my teeth and mouth problems when I complain; instead they focus on other issues	0.098	0.043	0.415	0.015	0.120	0.623
26. Treatment of mouth and teeth problems is too painful	0.051	0.407	0.197	0.211	−0.010	0.748
27. If I have a problem with my teeth or mouth, I do not tell anybody	0.107	0.081	0.090	0.362	0.054	0.840
28. I do nothing when I have pain in my mouth	0.227	0.002	0.236	0.501	−0.017	0.642
29. I do not know what to do when I have pain	0.084	0.190	0.185	0.571	0.017	0.597

COHIP, child oral health impact profile.

**TABLE 4:** The tool reliability analysis of the old and new sub-scales.

Items…In the past three months have you…	Item-test correlation	Item-rest correlation	Alpha if item deleted
**Oral health sub-scale (Alpha = 0.59)**			
1. Had pain in your teeth or toothache	0.65	0.38	0.47
2. Had bleeding gums	0.62	0.35	0.50
3. Had crooked teeth or spaces between your teeth	0.59	0.30	0.52
4. Had discoloured teeth or spots on your teeth	0.58	0.29	0.52
5. Had bad breath	0.58	0.28	0.52
**Self - Image sub-scale (Alpha = 0.56)**			
6. Felt that you were attractive			
7. Been confident because of your teeth and mouth			
**Social and emotional wellbeing Sub-scale (Alpha = 0.78)**			
8. Been teased, bullied	0.64	0.45	0.75
9. Been worried about what other people think	0.72	0.56	0.73
10. Been unhappy or sad	0.68	0.50	0.74
11. Avoided smiling or laughing	0.71	0.54	0.73
12. Felt that you look different	0.63	0.44	0.75
13. Felt worried or anxious	0.74	0.59	0.72
**School performance sub-scale (Alpha = 0.50)**			
14. Missed school for any reason because of your teeth			
15. Not wanted to speak/read out loud in class			
**Functional wellbeing sub-scale (Alpha = 0.56)**			
16. Had difficulty eating foods you would like to eat	0.72	0.42	0.41
17. Had trouble sleeping	0.71	0.41	0.41
18. Had difficulty saying certain words	0.61	0.26	0.55
19. Had difficulty keeping your teeth clean	0.61	0.27	0.54
**Dental access sub-scale (Alpha = 0.81)**			
20. Had a problem with getting dental care because the clinic or hospital is far	0.85	0.72	0.73
21. Had a problem with getting dental care because my parents or caregivers are usually at work	0.80	0.62	0.77
22. Had a problem with getting dental care because my family cannot afford	0.82	0.67	0.75
23. Had a problem with getting dental care because the clinics are closed	0.74	0.53	0.81
**Experience with dental care sub-scale (Alpha = 0.66)**			
24. At the dental clinic, they do not take my teeth and mouth complaints seriously	0.80	0.53	0.50
25. At the dental clinic, they do not address my teeth and mouth problems when I complain; instead, they focus on other issues	0.83	0.56	0.45
26. Treatment of mouth and teeth problems is too painful	0.71	0.356	0.72
**Coping sub-scale (Alpha = 0.60)**			
27. If I have a problem with my teeth or mouth, I do not tell anybody	0.71	0.33	0.59
28. I do nothing when I have pain in my mouth	0.77	0.43	0.44
29. I do not know what to do when I have pain	0.76	0.43	0.45

**Overall Cronbach-Alpha (for 29 items)**	**0.88**		

### Construct validity: Convergent and discriminant validity

Table 5 displays the different M-COHIP scores with several variables to test the discriminant and convergent validity hypothesis. The higher scores imply a poorer OHRQoL. The mean M-COHIP scores decreased with the better oral health self-rating and increased with self-report of toothache, active decay and caries experience (*p* < 0.001). The M-COHIP and/or OHRQoL scores for participants recruited from the school sites were higher than those at the HIV wellness site.

**TABLE 5:** Comparison of modified-child oral health impact profile by different variables for discriminant and convergent validity.

Variable	Sample	Modified-COHIP Score		*p*
	*N*	*n*	%	
**Oral health self-rating**				
Poor	66	67.55	20.51	*p* ≤ 0.001**
Average	119	63.7	16.94	-
Good	207	54.84	-	-
Total	392	-	-	-
**Toothache**				
Rarely	242	51.88	15.36	*p* ≤ 0.001**
Sometime	181	64.80	16.84	-
Always	70	74.44	18.87	-
Total	493	-	-	-
**Active decay**				
Decayed = 0	188	57.29	1.31	*p* ≤ 0.05*
Decayed > 0	309	61.06	1.06	-
Total	497	-	-	-
**Caries experience**				
DMFT = 0	160	57.03	1.47	*p* ≤ 0.05*
DMFT > 0	344	60.78	0.98	-
Total	504	-	-	-
**Sex**				
Male	298	60.46	1.03	> 0.05
Female	197	58.45	1.38	-
Total	495	-	-	-
**Site**				
HIV wellness	226	55.54	1.20	*p* ≤ 0.001**
School	278	63.59	1.08	-
Total	504	-	-	-

COHIP, child oral health impact profile.

* , *p* ≤ 0.05;

** , *p* ≤ 0.001.

Discriminant validity on groups was expected to demonstrate higher M-COHIP scores with a higher caries prevalence and with toothache reports. As depicted in Table 6 when adjusted for potential confounders such as age, sex, group, oral health self-rating and toothache, the caries prevalence and toothache were significant predictors of M-COHIP scores. The participants recruited from the school sites had a higher caries prevalence when compared with those in the wellness site, and the *p*-value was significant (*p* < 0.05). Age and sex were not covariates of modified COHIP scores in Johannesburg.

**TABLE 6:** Multivariate linear regression analysis for factors that may influence modified- child oral health impact profile among all participants.

Exposure variable	*N*	%	Regression coefficient	95% confidence interval	
				Lower	Upper
Age – Mean (s.d.)	15	2.0	−0.4	−1.1	0.3
Male	298	60.2	1	-	-
Female	197	39.8	−0.9	−4.1	2.4
Dental caries = 0	160	31.8	1	-	-
Dental caries > 0	344	68.3	4.5**	1.4	7.5
HIV wellness	226	44.8	1	-	-
School	278	55.2	3.3*	−0.0	6.6
Toothache-rarely	242	49.1	1	-	-
Sometimes	181	36.7	10.6***	7.3	13.9
Always	70	14.2	22.4***	17.9	26.9
Poor OH self-rating	66	116.8	1	-	-
Average OH self-rating	119	30.34	−1.16	−5.8	3.4
Good OH self-rating	207	52.8	−8.3***	−12.6	−4.1

s.d., standard deviation; HIV, human immunodeficiency virus.

* , *p* < 0.05;

** , *p* < 0.01;

*** , *p* < 0.001.

### Confirmatory factor analysis

**FIGURE 1:**
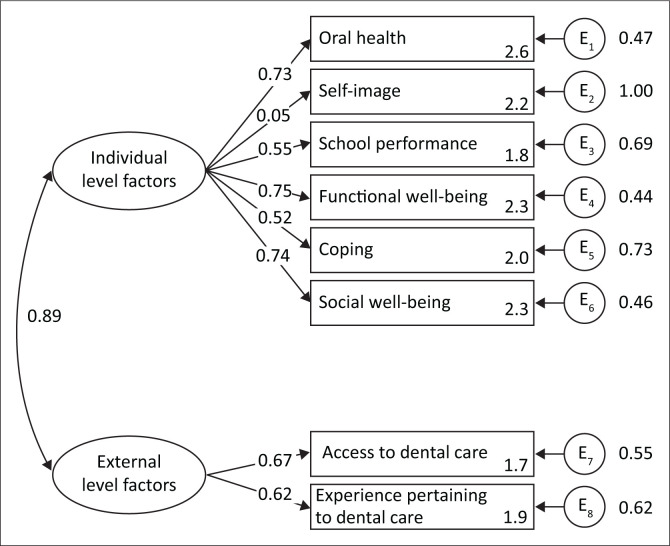
Confirmatory analysis model showing relationship between latent, observable factors and number of indicators or sub-scales relevant in the study setting.

Based on the theoretical model, the relationships between the three latent factors, viz. individual, external-level factors and social impact level factors were explored using the CFA model from structural equation modelling. The modelling was performed with the observed eight factors (Figure 1). The social impact latent factor was not included as it had only one sub-scale associated with it (socio-emotional well-being).

The CFA results of the overall fit for the source of the model are shown as χ^2^ test (*p* < 0.001), RMSEA 0.05 with corresponding 90% confidence interval of 0.04–0.07, SRMR = 0.034, TLI = 0.959, CFI = 0.972. The overall fit indices predicted a good model. The final model had two latent factors, viz. individual and external-level factors related to one another (Figure 1).

The school performance and coping sub-scale were marginally correlated with the loading of 0.55 and 0.52, respectively. The eighth, Social Image sub-scale had the weakest convergence with loading less than 0.5 (0.44).

## Discussion

The 29-item, South African version exhibited acceptable properties. The M-COHIP is the result of the contextual findings of a prior qualitative inquiry, which suggested the addition of three more sub-scales to capture the relevant factors important in the setting.^14^ These new sub-scales ‘Dental care access’, ‘Experience with dental care’ and ‘Coping’ were added, and the final tool displayed very good reliability and validity.

Cronbach’s alpha revealed an acceptable level of internal consistency and reliability ranging from moderate (0.5) to (0.8) for all eight sub-scales and was excellent for all the 29 items at 0.88. Its overall score is similar to the one in the Ahn et al. study in their Korean sample of 0.88,^18^ El Osta et al. found 0.88 in their Caledonian sample^19^ and Li et al. found 0.88 in their Chinese sample. Elsewhere, Broder found a comparable score of 0.91 among the Canadian children’s participants.^13^ The developers of Broder et al. also shortened the original 34-item tool to the Shortened Form-COHIP,^13^ where Agnew et al. in Australia and Arheniam et al. in Libya adapted and used it. The latter authors found the Cronbach’s- alpha of 0.9 and 0. 84, respectively.^17,20^

Our study found the M-COHIP score of mean 59.6 (18.42) and median score of 58 (IQR: 46–72). When reported by site, the HIV wellness group score was 55.5 (1.2), while that for the school participants was 62.9 (1.09). When they applied a shortened form of the COHIP tool, Arheiam and colleagues scored their participants 61.1 using COHP-SF, while a Chinese study reported 62.2. Self-image as a sub-scale performed well with highest correlates compared with this study where it had an average Cronbach’s alpha score of 0.6. However, school performance had the lowest alpha of 0.5.^17^ The two items measuring self-image in our study, and the original Broder study were the only positively worded phrases out of negatively worded statements.

When it comes to the Self-Image dimension – regardless of the correlation, the reliability was high (0.6) enough for the sub-scale to be retained as posited by Worthington and Whittaker in the theory of scale development.^21^

Poor oral health self-rating inversely correlated with higher modified COHIP scores confirming the ability of the scale to differentiate the behavioural manifestations; thus, convergent validity was correlated with oral health self-rating. The impacts decreased with lower scores, implying a good OHRQoL score and good global rating. The latter was also reported by several studies where poorer self-rated oral health interconnected with poor OHRQoL.^19,20^ The relatedness in the correlation matrix detected gives evidence of the validity of the estimated item through correlations of the overall modified COHIP score, and the sub-scale with the perceived self-rating by participants. The results are comparable to Agnew et al., where the same proxy elements for the positive global rating were related to their COHIP scores.^20^

Furthermore, less decay, mild toothache and access to care for diseases may have contributed to the overall better mean scores between two groups of adolescents in the two research groups, depicting a good discriminant validity. Dental caries prevalence was generally high at 62%, among this combined cohort of adolescents. However, ALHIV were receiving care from the wellness centre. The results showed a lesser caries prevalence and a slightly more treatment component care seen by extraction services (M) and the teeth restored (F) compared with the participants recruited from the school sites. The modified – COHIP tool, when applied to this setting, among these participants, could differentiate the OHRQoL scores in the two groups. The pain symptom was congruent with the dental caries status. This is in the same tone with what Arheniam and co-authors’ results showed that active decay had poorer OHRQoL.^17^ The adolescents in the school site reported higher modified COHIP (62.88) than the participants recruited from the wellness site (55.54).

Initial EFA led to five tentative dimensions compared with the four dimensions in Arheniam et al. study. The authors suggest that during scale development or adaptation, the most rational and logical approach is to conduct EFA before confirmation by CFA.^21^ This led to five original and new sub-scales to constitute the 8-sub-scale M-COHIP. The similar process by Arheniam et al. in their Arabian version the initial EFA identified a four-factors solution even though the original had three factors.^17^ The EFA is a fluid and dynamic elimination and revision stage used to cluster items. Using EFA as an item selection process may yield various outcomes when the items are both formative and reflective. The number of factors may range from two to nine.^21^ Eventually, a five-factor structure was adopted after two factors with eigenvalue over 1 were revealed and the parallel analysis suggested six factors.

Confirmatory factor analysis was used to validate the relationships following the EFA. The relationships were also based on the priori theory of the relationship between individual and external factors influencing the OHRQoL. According to Kline,^22^ the standardised loadings (*r*^2^) should be at least 0.70 to display good convergent validity. The acceptable cut-off points are from 0.5. The dental care access (0.67) and the dental care experiences (0.62) showed good convergence to the external level latent factor. Likewise, the oral health, functional well-being and the socio-emotional well-being had the highest convergence (*r*^2^ > 0.70) to the individual latent factor.

This study is not without limitations; the self-report nature of OHRQoL is subjective and depends on the recall ability of the participants. Scholars deem reliability and validity as incremental and unending processes. Thus, the reported psychometric properties of our tool are correct for this setting and participants under the described circumstances. Test-retest assessment, which is an adjunct to measures of reliability, was not possible in the setting because of the structure of the HIV centre participant burden. However, the robust validity measures employed showed good results. The results are applicable to the study participants and sites.

## Conclusion

The modified-COHIP displayed good psychometric properties. Furthermore, their OHRQoL, regardless of HIV status, was impacted by the dental caries status. The finding supports the notion that, with dental caries, the predisposing factors are largely similar regardless of the HIV infection. This study showed that those with higher decay component were mostly in schools; hence, being selected from the school was a predictor to having a poorer OHRQoL. This comparative study found that for this population, being on treatment at the HIV wellness site, might be protective against caries. The overall adolescents’ OHRQoL scores were related to the high untreated caries, toothache reports, a poor self-rated oral health and being in schools. The result of this study provides a reliable questionnaire for use by researchers and clinicians while measuring patient-reported quality of life outcomes related to oral diseases in the programmes. These reported outcomes are part of the overall quality care appraisals.
